# Investigation of the ultrasound-induced collapse of air bubbles near soft materials

**DOI:** 10.1016/j.ultsonch.2023.106723

**Published:** 2023-12-09

**Authors:** Armand Shams, Saeed Bidi, Manolis Gavaises

**Affiliations:** a School of Science and Technology, City, University of London, UK; b Institut Jean le Rond d’Alembert, Sorbonne Université and CNRS UMR 7190, F-75005 Paris, France

**Keywords:** Fluid-structure interaction, Eulerian hyperelasticity, Ultrasounds, Bubble dynamics, Cavitation, Diffuse interface model

## Abstract

A numerical investigation into the ultrasound-induced collapse of air bubbles near soft materials, utilizing a novel multi-material diffuse interface method (DIM) model with block-structured adaptive mesh refinement is presented. The present work expands from a previous five-equation DIM by incorporating Eulerian hyperelasticity. The model is applicable to any arbitrary number of interacting fluid and solid material. A single conservation law for the elastic stretch tensor enables tracking the deformations for all the solid materials. A series of benchmark cases are conducted, and the solution is found to be in excellent agreement against theoretical data. Subsequently, the ultrasound-induced bubble-tissue flow interactions are examined. The bubble radius was found to play a crucial role in dictating the stresses experienced by the tissue, underscoring its significance in medical applications. The results reveal that soft tissues primarily experience tensile forces during these interactions, suggesting potential tensile-driven injuries that may occur in relevant treatments. Moreover, regions of maximal tensile forces align with tissue elongation areas. It is documented that while early bubble dynamics remain relatively unaffected by changes in shear modulus, at later stages of the penetration processes and the deformation shapes, exhibit notable variations. Lastly, it is demonstrated that decreasing standoff distances enhances the interaction between bubbles and tissue, thereby increasing the stress levels in the tissue, although the behavior of the bubble dynamics remains largely unchanged.

## Highlights

• A novel multi-material diffuse interface method model with block-structured adaptive mesh refinement is presented.
• The soft material predominantly experiences tensile forces compared to compressive or shear forces, suggesting that injuries are mainly tensile-driven.
• The bubble radius and the standoff distance play pivotal roles in the stresses experienced by the soft material, emphasizing their importance in medical applications.

## Graphical abstract

**Figure:**
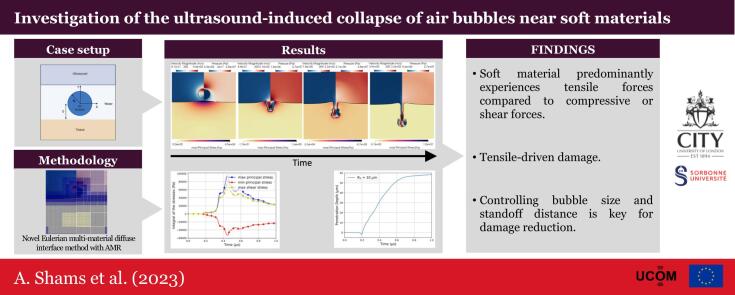

## 1 Introduction

Bubbles and ultrasounds have been used in a wide variety of applications in medicine [1]. In medical diagnostics, sonography has utilized ultrasounds for decades. The recent introduction of Ultrasound Contrast Agents (UCAs), microbubbles encased in lipid or protein layers, has enhanced imaging clarity [2]. Unlike traditional bubbles, these UCAs emit strong signals when exposed to an ultrasound field, offering targeted visualization around them. In therapeutic contexts, Ultrasonic Cavitation (UC) has made significant strides in surgery and drug delivery. High-Intensity Focused Ultrasound (HIFU) employs specific sound waves to thermally target tumors in cancer treatment [3]. Yet, there are safety concerns, including potential tissue damage and unintended tissue heating [4]. A newer approach, histotripsy, circumvent these issues by mechanically ablating tissue without heat [5]. Cavitation techniques, such as Extracorporeal Shockwave Lithotripsy (ESWL), have been in use since the 80 s for kidney stone treatment. For drug delivery, UC synergizes with UCAs to enhance drug absorption in tissues, a process termed sonoporation [6]. The mechanism, involving pore formation from various stimuli, remains intricate and not entirely understood [7]. Under certain conditions, bubbles exert mechanical stress on the membrane, leading to permeabilization [8], [9]. Higher acoustic pressures can result in microjets that breach the cell membrane [10], and shock-membrane interactions further contribute [11].

Understanding the complex dynamics between the bubble and the surrounding tissue could enhance the therapeutic potential of these techniques. However, the multi-physics character of these flows makes them difficult to be solved using numerical methods. The Arbitrary Lagrangian-Eulerian (ALE) and Immersed Boundary (IB) methods stand out as prominent numerical techniques in fluid–structure interactions (FSI) [12], [13], [14]. The ALE method blends Lagrangian and Eulerian descriptions by deforming the computational mesh in response to structural movement, thereby offering a flexible approach that accommodates both fluid and structural dynamics [15]. On the other hand, the IB method employs separate grids for the fluid and the solid, wherein the structure is “immersed” in the Eulerian fluid grid. The interaction between the fluid and solid is then prescribed by boundary conditions at the interface [16]. However, both methods exhibit limitations, especially in cases of large deformations. For the ALE method, large deformations can lead to mesh tangling and reduced mesh quality, which consequently demands frequent remeshing [17]. Ensuring mesh quality and preventing tangling or distortion, especially under significant deformations, are major challenges. The IB method, while being versatile for complex geometries, can face challenges in accurately representing the structure-fluid interface during large deformations. The inherent smearing of the interface and the potential for non-physical oscillations in the vicinity of the immersed boundary can sometimes affect the solution quality [18]. In contrast, Eulerian methods are more suitable for simulating large deformations as they decouple material and spatial coordinates. Recent advancements in the formulation of elasticity in the Eulerian frame [19], [20], [21], [22], [23], [24] facilitate its application to FSI problems when combined with a sharp [25] or diffuse interface method [19] (DIM) to include multiple materials. Various works have been published using Eulerian elasticity with sharp interface methods, utilizing the Ghost Fluid Method (GFM) [25], [26], [27] or the cut-cell method [28], [29], [30]. While these approaches preserve sharp interfaces through complex reconstructions and mixed-cell algorithms they are challenging to implement especially in the case of adaptive mesh refinement (AMR) and introduce non-conservative terms in the case of GFM. Alternatively, a number of DIM have been published for multi-fluid flows [31], [32], [33], [34], [35], [36], [37] applied to bubble dynamics [38], [39], [40], droplet fragmentation [35], complex thermodynamics [41], [42], [43], and cavitation sub-grid models [43], [44], [45], [46], [47], [48]. In fact, the multi-fluid pressure relaxation model of [33] was later used in [19], [49], [50] to incorporate Eulerian hyperelasticity and plasticity However, tracking deformation gradients for numerous solid materials is computationally prohibitive. A single deformation tensor for multiple materials was proposed in [51], reducing the number of equations required to be solved, but sometimes causing significant errors at interfaces of large density gradients. A single conservation law on the stretch tensor to track the deformations for any number of solids was proposed in [22], which is associated with the Allaire DIM [31]. It was then applied to the FSI of reactive fluids and elastoplastic solids [52] as well as sliding and void opening problems [53]. The Allaire model used in those publications presents a significant shortcoming. It was shown in [38] that this DIM is incapable of capturing bubble dynamics relevant to the conditions examined in the present work, due to its thermodynamic incompatibility.

A few numerical studies have been published on the topic of bubble collapse or oscillation near soft materials. In [54] investigated bubble oscillations near a fluid–fluid interface utilizing a Boundary Integral Method (BIM), highlighting that depending on the density ratio, bubbles could either gravitate towards or be repelled from the interface. The authors of [55] expanded the previous work by adding elasticity to the interface, unveiling the emergence of mushroom-shaped bubbles. This phenomenon was experimentally substantiated in [56]. Following in [57] investigated shock-induced bubble jetting in proximity to viscous fluids, concluding that increased tissue viscosity can significantly reduce jet penetration depth. In [58] studied shock bubble interaction near soft and rigid boundaries modeled as fluids during lithotripsy using an improved Ghost Fluid Method (GFM). The impulse from the bubble's collapse was linked to tissue displacement, potentially causing tissue damage or stone fragmentation. In [40] studied the potential injury mechanisms in shockwave lithotripsy in blood vessels utilizing a multi-fluid DIM, discovering that as bubble confinement increases, so do the pressures and deformations on the vessel wall. In [59] utilized a free Lagrangian method to investigate the impingement of high-speed liquid jets resulting from shock-induced collapsing bubbles. Their findings showed that these jets exerted such significant compression on aluminum that it led to both pitting and plastic deformation. The authors of [60] investigated bubble shapes and maximum jet velocity when subject to an ultrasound forcing near different soft materials using BEM with linear elasticity. In [61] studied the ultrasonic forcing of a UCA bubble above a tissue layer with rigid backing using a BIM. The re-expansion of the toroidal bubble could separate the tissue layer from the rigid backing, a mechanism identified as “peeling”. In [62] utilized a two-dimensional Finite Element Method (FEM) to analyze bubble–blood–vessel interactions, showing that vessel constraints can shift a bubble's resonance frequency, causing asymmetric oscillations and inducing potentially damaging shear stress on the vessel wall. The study emphasized the role of the bubble's resonance frequency and ultrasound contrast agent shell elasticity in these dynamics. In [63] utilized a BEM to investigate microbubble dynamics in elastic micro-vessels under ultrasound forcing. Their findings highlighted that when the bubble and vessel's radii are comparable, the ultrasound forcing can cause the bubble to elongate within the vessel, forming counter jets that deform the vessel wall. In [64] investigated the impact of a shockwave on a bubble near various solid materials and the effect of the acoustic impedance on the shockwave emissions and liquid jet strength. They utilized a partitioned approach where the fluid was solved using a compressible multi-fluid solver and the solid using a FEM solver. Lastly, the work of [65] utilized an Eulerian multi-material DIM [49] with AMR to investigate the hock-induced bubble collapse near solid materials during lithotripsy. Their findings highlight the importance of the bubble standoff distance on the shapes of the bubble and of the tissue. While these studies provide valuable insight into the bubble dynamics: collapse pressure, liquid jet velocities, and shape of the bubble; very little focus if any has been placed on the stresses developed in soft materials.

In the present work, the aim is to provide a comprehensive study of the ultrasound-induced collapse near soft materials with a focus on the mechanical loads experienced by the material. Firstly, a novel model based on the five-equation DIM [37] is outlined, augmented by the kinematic equations of the stretch tensor [22]. Unlike the previously published Eulerian hyperelasticity models of [19], [23], [50], [66] the current model only uses a single kinematic equation to track deformations. Moreover, these previous publications utilized the HLLC Riemann solver where the shear waves were modeled as a contact discontinuity. Therefore, the shear waves are unnecessarily diffusive. Here, the HLLD Riemann solver presented in [22], [67] was utilized, which introduces a family of slow waves used to model the shear waves. Furthermore, unlike [22], the DIM used in the present work is thermodynamically compatible and thus capable of capturing bubble dynamics. A block-structured AMR with local time-stepping was utilized to accurately solve the different scales of the multi-material flow and preserve the sharpness of the interfaces and waves. Secondly, a comprehensive analysis of mechanical loads experienced by the soft material is presented by visualizing and integrating the maximum and minimum principal stress and the maximum shear stress. Finally, the potential for material failure is identified by looking at the highest maximum principal stress areas. Thus, the contributions of the present work are threefold: (a) a novel DIM model for multi-material simulations with AMR, (b) an understanding of the mechanical loading of the tissue under ultrasound-induced collapse, and (c) the potential areas of material failure and thus, tissue injury.

The remainder of the paper is organized as follows. The governing equations, the thermodynamics closure, and the constitutive model for the solid are presented in Section 2. The numerical methods utilized are described in Section 3. Benchmark cases to assess the accuracy of the model are compared with the present contribution in Section 4. In Section 5, a detailed description of the ultrasound-induced bubble collapse near a soft material is provided, and then an analysis of the effect of the shear modulus, the initial bubble radius, and the standoff distance on the deformation is presented. Finally, in Section 6 the findings of the paper are summarized.

## 2 Governing equations

The non-conservative seven equation model of [68] is known to be the most general and complete diffuse interface model able to capture complex wave patterns. Indeed, this full disequilibrium model considers each phase to have its own pressure, velocity and temperature. The time scales of these variables at equilibrium conditions are modeled by source terms. However, since the time scales for equilibration are small, it leads to stiff source terms, making its numerical resolution challenging. To overcome this, in this work, a reduced model is used by applying stiff mechanical relaxation leading to the well-known five-equation model of [37] with a single pressure, velocity, and deviatoric strain in the mixture regions. The multi-component flow model is extended with a kinematic equation for the elastic stretch tensor incorporating Eulerian hyperelasticity. In the limit of 2 materials, the model results in a non-conservative volume faction equation, two mass, one momentum, one energy conservation equations, and in addition, nine non-conservative elastic stretch equations. The resulting model can accurately simulate fluid–structure interactions for any number of material interfaces and can exhibit complex wave patterns where both acoustic and stress waves are captured. In this paper, the focus is placed on ultrasound-driven bubble collapse near soft materials at time scales where inertial forces dominate. Hence, the effect of surface tension, viscosity, mass transfer, and phase transition are neglected, see [69], [70], [71] for justification. For l=1,⋯,N materials:

(1) $$\frac{\partial }{\partial t}\left(\begin{array}{c} \alpha_{\left(l\right)}\\ \alpha_{\left(l\right)}\rho_{\left(l\right)}\\ \begin{array}{c} \rho u_{i}\\ \begin{array}{c} \rho E\\ {\overset{¯}{V}}_{\mathrm{ij}}^{e} \end{array} \end{array} \end{array}\right)+\frac{\partial }{\partial x_{k}}\left(\begin{array}{c} \alpha_{\left(l\right)}u_{k}\\ \alpha_{\left(l\right)}\rho_{\left(l\right)}u_{k}\\ \begin{array}{c} \rho u_{i}u_{k}-\sigma_{\mathrm{ik}}\\ \begin{array}{c} \rho Eu_{k}-u_{i}\sigma_{\mathrm{ik}}\\ {\overset{¯}{V}}_{\mathrm{ij}}^{e}u_{k}-u_{i}{\overset{¯}{V}}_{\mathrm{kj}}^{e} \end{array} \end{array} \end{array}\right)=\left(\begin{array}{c} \left(\alpha_{\left(l\right)}+K_{\left(l\right)}\right)\frac{{\partial u}_{k}}{\partial x_{k}}\\ 0\\ \begin{array}{c} 0\\ \begin{array}{c} 0\\ \frac{2}{3}{\overset{¯}{V}}_{\mathrm{ij}}^{e}\frac{{\partial u}_{k}}{\partial x_{k}}-u_{i}\frac{\partial {\overset{¯}{V}}_{\mathrm{kj}}^{e}}{\partial x_{k}} \end{array} \end{array} \end{array}\right),$$

where the scalar fields $\alpha_{i}$, $\rho_{i}$, $u_{i}$, E are the volume fraction, the density, the velocity, and the total energy, σ the stress tensor, ${\overset{¯}{V}}^{e}$ the symmetric left unimodular stretch tensor. The compression and expansion of each phase in the mixture region are modeled by $K_{\left(l\right)}\frac{{\partial u}_{k}}{\partial x_{k}}$ where:

(2) $$K_{\left(l\right)}=\alpha_{\left(l\right)}\left(\frac{\rho c_{p}^{2}}{\rho_{\left(l\right)}c_{\left(l\right)}^{2}}-1\right)$$

with the pressure equilibrium speed of sound [34], a generalization of Wood’s speed of sound expressed as:

(3) $$c_{p}={\left(\rho \sum_{l=1}^{N}\frac{\alpha_{\left(l\right)}}{\rho_{\left(l\right)}c_{\left(l\right)}^{2}}\right)}^{-\frac{1}{2}}$$

The mixture total energy is:

(4) $$E=e+\left|u\right|/2$$

where e is the mixture specific internal energy and u is the velocity vector. The following mixture rule for the internal energy applies:

(5) $$e=\sum_{l=1}^{N}Y_{\left(l\right)}e_{\left(l\right)}\left(\rho_{\left(l\right)},p,\overset{¯}{B}\right)$$

where $e_{\left(l\right)}$ are the specific internal energies of each phase, $Y_{\left(l\right)}$ are the mass fractions of each phase, $\overset{¯}{B}={\overset{¯}{F}}^{T}\overset{¯}{F}$ is the unimodular part of the left Cauchy Green strain tensor and $\overset{¯}{F}$ is the unimodular deformation tensor. The mass fractions of each phase are given by:

(6) $$Y_{\left(l\right)}=\frac{\alpha_{\left(l\right)}\rho_{\left(l\right)}}{\rho }$$

The specific internal energy e for each material is defined by an equation of state (EoS) and a constitutive law where the hydrodynamic and elastic contributions are separated [23] with the following form:

(7) $$e_{\left(l\right)}\left(\rho_{\left(l\right)},p,\overset{¯}{B}\right)=e_{\left(l\right)}^{h}\left(\rho_{\left(l\right)},p\right)+e_{\left(l\right)}^{s}\left(\rho_{\left(l\right)},\;\overset{¯}{B}\right)$$

The hydrodynamic part $e_{\left(l\right)}^{h}$ depends only on the density and pressure while the elastic part $e_{\left(l\right)}^{s}$ depends on the density and strain tensor. A major advantage of this additive decomposition is the decoupling between the two contributions. The pressure is only defined by the hydrodynamic energy and the deviatoric stress tensor is only defined by the elastic energy. The stiffened gas EoS is used for the hydrodynamic energy:

(8) $$p_{\left(l\right)}=\left(\gamma_{\left(l\right)}-1\right)\rho_{\left(l\right)}e_{\left(l\right)}-\gamma_{\left(l\right)}p_{\infty ,\left(l\right)}$$

where $\gamma_{\left(l\right)},$ and $p_{\infty ,\left(l\right)}$ are parameters of the EoS. The speed of sound of each material is defined as:

(9) $$c_{\left(l\right)}^{2}=\frac{\gamma_{\left(l\right)}\left(p+p_{\infty ,(l)}\right)}{\rho_{\left(l\right)}}+\frac{4}{3}\frac{\mu_{\left(l\right)}}{\rho_{0\left(l\right)}}$$

The elastic energy is subject to the choice of the strain energy density function. The Neo-Hookean model was chosen here as it is a popular non-linear constitutive relationship used in biomedical applications to model tissue:

(10) $$e_{\left(l\right)}^{s}\left(\rho_{\left(l\right)},\bar{B}\;\right)=\frac{\mu_{i}}{2\rho_{0i}}\left({\bar{I}}_{1}-3\right)$$

where ${\overset{¯}{I}}_{1}$ is the first invariant of the unimodular left Cauchy Green strain tensor defined as:

(11) $${\overset{¯}{I}}_{1}=tr\left(dev\left(\overset{¯}{B}\right)\right)$$

with $dev\left(\overset{¯}{B}\right)=\overset{¯}{B}-tr\left(\overset{¯}{B}\right)I$ is the matrix deviator and $\mathrm{tr}(\overset{¯}{B})$ is the trace. The Cauchy stress tensor is derived from the constitutive law:

(12) $$\sigma =\frac{2}{J}B\frac{\partial W}{\partial B}$$

where J=det(F) is the Jacobian of the deformation tensor. For a Neo-Hookean constitutive law, the Cauchy stress tensor can be expressed as follows:

(13) $$\sigma =-pI+\frac{\rho \mu }{\rho_{0}}dev\left(\overset{¯}{B}\right)$$

where p is the mixture pressure, ρ is the mixture density, $\rho_{0}$ is the initial mixture density of the materials and μ is the mixture shear modulus. The above formulation of the stress tensor allows modeling of both solids and fluids. For the latter, the shear modulus is zero, thus resulting in a spherical stress tensor and no elastic energy contribution. The mixture density is defined according to the mixture rule:

(14) $$\rho =\sum_{l=1}^{N}\alpha_{\left(l\right)}\rho_{\left(l\right)}$$

The saturation constraint equation is required to evaluate the volume fraction of the phases:

(15) $$\sum_{l=1}^{N}\alpha_{\left(l\right)}=1$$

## 3 Numerical methods

The system of basic equations described above is hyperbolic and can be cast into semi-conservative form to be solved by a Godunov-type scheme [72]:

(16) $$\frac{\partial q}{\partial t}+\frac{\partial F^{k}}{\partial x_{k}}=s_{\mathrm{non}-cons}+s_{g}$$

where q is the vector of state variables, $F^{k}$ are the vectors of fluxes in the respective directions x, y, z, $s_{\mathrm{non}-cons}$ is the vector of non-conservative source terms, and $s_{g}$ is the vector of geometrical source terms. The vector of state variables, vectors of fluxes, and non-conservative source terms are expressed as:

(17) $$q=\left(\begin{array}{c} \alpha_{\left(l\right)}\\ \alpha_{\left(l\right)}\rho_{\left(l\right)}\\ \begin{array}{c} \rho u_{i}\\ \begin{array}{c} \rho E\\ {\overset{¯}{V}}_{\mathrm{ij}}^{e} \end{array} \end{array} \end{array}\right),\;F^{k}=\left(\begin{array}{c} \alpha_{\left(l\right)}u_{k}\\ \alpha_{\left(l\right)}\rho_{\left(l\right)}u_{k}\\ \begin{array}{c} \rho u_{i}u_{k}-\sigma_{\mathrm{ik}}\\ \begin{array}{c} \rho Eu_{k}-u_{i}\sigma_{\mathrm{ik}}\\ {\overset{¯}{V}}_{\mathrm{ij}}^{e}u_{k}-u_{i}{\overset{¯}{V}}_{\mathrm{kj}}^{e} \end{array} \end{array} \end{array}\right),s_{\mathrm{non}-cons}=\left(\begin{array}{c} {(\alpha }_{\left(l\right)}+K_{\left(l\right)})\frac{{\partial u}_{k}}{\partial x_{k}}\\ 0\\ \begin{array}{c} 0\\ \begin{array}{c} 0\\ \frac{2}{3}{\overset{¯}{V}}_{\mathrm{ij}}^{e}\frac{{\partial u}_{k}}{\partial x_{k}}-u_{i}\frac{\partial {\overset{¯}{V}}_{\mathrm{kj}}^{e}}{\partial x_{k}} \end{array} \end{array} \end{array}\right)$$

The geometrical source term is defined as:

(18) $$s_{g}=-\frac{\beta }{r}\left(\begin{array}{c} 0\\ \alpha_{\left(l\right)}\rho_{\left(l\right)}u_{r}\\ \begin{array}{c} \rho u_{r}^{2}-\sigma_{\mathrm{rr}}+\sigma_{\theta \theta }\\ \rho u_{r}u_{z}-\sigma_{\mathrm{rz}}\\ \begin{array}{c} \rho Eu-\left({u_{r}\sigma }_{\mathrm{rr}}+u_{z}\sigma_{\mathrm{rz}}\right)\\ \frac{1}{3}{\overset{¯}{V}}_{\mathrm{ij}}^{e}u_{r}-\delta_{i\theta }{\overset{¯}{V}}_{\mathrm{ij}}^{e}u_{r} \end{array} \end{array} \end{array}\right)$$

where β=1 when the coordinate system is axisymmetric and β=2 when the coordinate system is spherical. After spatially integrating the conservation law (1 6 ) by taking the volume integral and applying the divergence theorem we obtain the finite volume discretization:

(19) $$\frac{dq_{i,j,k}}{\mathrm{dt}}=\frac{1}{\Delta x_{i}}\left[F_{i-1/2}^{x}-F_{i+1/2}^{x}\right]+\frac{1}{\Delta y_{j}}\left[F_{j-1/2}^{y}-F_{j+1/2}^{y}\right]+\frac{1}{\Delta z_{k}}\left[F_{k-1/2}^{z}-F_{k+1/2}^{z}\right]+s_{i,j,k}$$

The cell-averaged vector of state variables and the face-averaged vectors of fluxes are defined as:

(20) $$q_{i,j,k}=\frac{1}{V_{i,j,k}}{∭}_{I_{i,j,k}}q\left(x,y,z,t\right)dxdydz$$

(21) $$F_{i+1/2,j,k}=\frac{1}{\Delta y_{j}\Delta z_{k}}\iint_{A_{i+1/2,j,k}}F\left(x,y,z,t\right)dydz$$

The fluxes at the cell faces are computed using an approximate Riemann solver. The Harten, Lax, and van Leer (HLL) Riemann solver and its derivatives, are solutions for wave propagation problems. The HLL method uses a wave configuration that separates three constant states and assumes that wave speeds can be estimated from these initial states. The HLL-contact (HLLC) scheme expands on this by handling four states separated by a contact discontinuity. However, this does not accurately represent the conditions in solids where additional families of characteristics related to shear wave speeds exist. The HLLD approach used here [22], [67] accounts for this by introducing the concept of multiple slower waves between the fastest waves and the contact discontinuity. The piece-wise linear MUSCL reconstruction was used on the primitive variables to reconstruct the states at the faces to avoid spurious oscillations at the material interfaces [39], [73].

### 3.1 Temporal integration

After the spatial derivatives are approximated, a semi-discrete system composed of ordinary differential equations in time is obtained where a two-step time integration was applied [74] resulting in a second-order temporal integration:

(22) $$q_{i,j,k}^{n+\frac{1}{2}}=q_{i,j,k}^{n}+\frac{1}{2}\Delta tL\left(q_{i,j,k}^{n}\right)$$

(23) $$q_{i,j,k}^{n+1}=q_{i,j,k}^{n}+\Delta tL\left(q_{i,j,k}^{n+\frac{1}{2}}\right)$$

where L is the right-hand side of ( 1 9 ), and n is the timestep index. The timestep size Δt is determined using the acoustic-based Courant–Friedrichs–Lewy (CFL) number.

### 3.2 Adaptive mesh refinement

The numerical methods used in this work have been developed and implemented within the AMReX framework [75], [76]. This is a C++ based framework that allows for solving partial differential equations using block-structured adaptive mesh refinement algorithms. In [77] structured adaptive mesh refinement (AMR) method was used to solve the hyperbolic system detailed below utilizing adaptive refinement in space and time. The computational mesh is broken down into logically rectangular sub-grids where cells have the same resolution. The sub-grids, also called patches, are organized into a hierarchy of embedded levels. The lowest resolution grids at level 0 spans across the entire computational domain. A refinement criterion is utilized for generating finer levels from the coarser ones at a user-defined interval of timesteps. Cells at level l are $r_{l}$-times finer than at level l-1 with $\Delta x_{l}=\Delta x_{l-1}/r_{l}$ and $\Delta t_{l}=\Delta t_{l-1}/r_{l}$ where $r_{l}\in N$, $r_{l}\geq 2$ for l>0 and $r_{0}=1$. A subcycling-in-time approach was utilized where the coarse grid solution is advanced in time ignoring the finer levels. Then the finer levels are advanced recursively while using the coarser levels as boundary conditions. At the coarse–fine interfaces, the ghost cells are determined using conservative linear interpolation in space and time. The solution on the coarse grids needs to be corrected by the finer levels to ensure global conservation. Therefore, once a fine level has reached the same physical time as the coarser level a synchronization step is applied. The volume averages of the coarse cells are corrected to be equal to the volume averages of the finer cells. Additionally, the area and time-weighted fluxes at the coarse–fine interfaces are also corrected by the sum of the fine fluxes.

## 4 Validation and verification

In this section, verification studies against well-known benchmark cases are presented. To showcase the numerical model's ability to capture bubble dynamics, we simulate the collapse of a spherical gas bubble in an infinite water medium at different pressure ratios. Additionally, to evaluate the accuracy of the model to predict fluid–solid interactions and wave transmission across material interfaces, a semi-analytical wave propagation case is conducted. Finally, the bubble-ultrasound interaction is validated through an ultrasound-bubble-rigid wall interaction case.

### 4.1 Spherical bubble collapse

A common benchmark case for multi-phase flow solvers is the comparison against the Keller-Miksis equation [38], [78], [79]. The effects of surface tension and viscosity are ignored. Two cases are presented, corresponding to a low $p_{f}/p_{b}=20$ and a high initial pressure ratio $p_{f}/p_{b}=353;$ for the former a grid convergence study was also performed. In both cases the initial bubble radius $R_{0}=1mm$ where the domain size $L=50R_{0}$. Spherical coordinates with β=2 as detailed in Eq (18) and a base mesh of 512 cells in the radial direction have been employed. A grid convergence study was conducted, and it was found that 2 levels of refinement is sufficient to match the Keller-Miksis solution. At the center of the bubble, a symmetry boundary condition was used. At the far-field, an outflow boundary condition was imposed. The pressure is initialized uniformly inside the bubble and gradually increases in the water medium according to [38]:

(24) $$p\left(R\right)=p_{f}+\frac{R_{0}}{R}\left(p_{b}-p_{f}\right)$$

where $p_{f}$ is the far-field pressure, R is the radial coordinate and $p_{b}$ is the bubble pressure. The initial water density is $\rho_{\mathrm{water}}=998.2kg\cdot m^{-3}$ and the stiffened gas parameters are $\gamma_{\mathrm{water}}=4.4$ and $p_{\infty ,water}=6\cdot 10^{8}Pa$. The initial air density is $\rho_{\mathrm{air}}=1.225kg.m^{-3}$ and the stiffened gas parameters are $\gamma_{\mathrm{water}}=1.4$ and $p_{\infty ,air}=0Pa$ which results in considering an ideal gas. For the first case, an initial low-pressure ratio of $p_{f}/p_{b}=20$ is considered. The bubble radius is normalized by its initial radius $R_{0}$ and time is normalized by the Rayleigh collapse time [79] expressed as:

(25) $$t_{c}=0.915R_{0}\sqrt{\frac{\rho_{\mathrm{water}}}{p_{f}}}$$

**Fig. 1:**
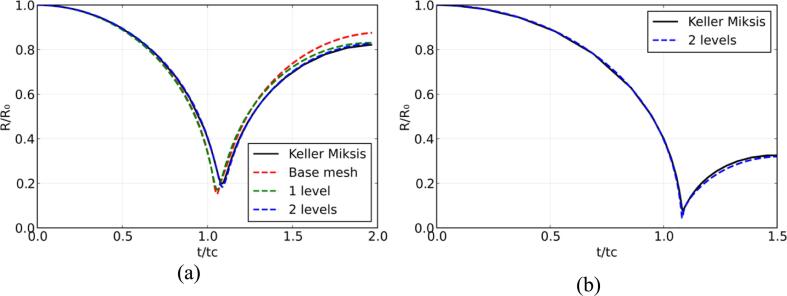
Temporal evolution of the normalized bubble radius over normalized time for both spherical bubble collapse cases. (a) Low pressure ratio $p_{f}/p_{b}=20$ with 3 different AMR grids. (b) High-pressure ratio case $p_{f}/p_{b}=353$ with 2 levels of refinement.

The Keller Miksis solution was solved using Julia [80] and the DifferentialEquations.jl [81] package with Rosenbrock temporal integration. In Fig. 1, the bubble radius evaluation is depicted for both cases; excellent agreement was found. The low-pressure case was run with the base grid, one level, and two levels of refinement. The latter was found to fit the solution and hence chosen for the high-pressure case. The rebound and minimum radius observed in the high-pressure ratio case are smaller due to the compressibility effects. In fact, the present results show the capability of the method to capture compression and expansion rates accurately.

### 4.2 Semi-analytical wave transmission across a fluid/solid interface

**Fig. 2:**
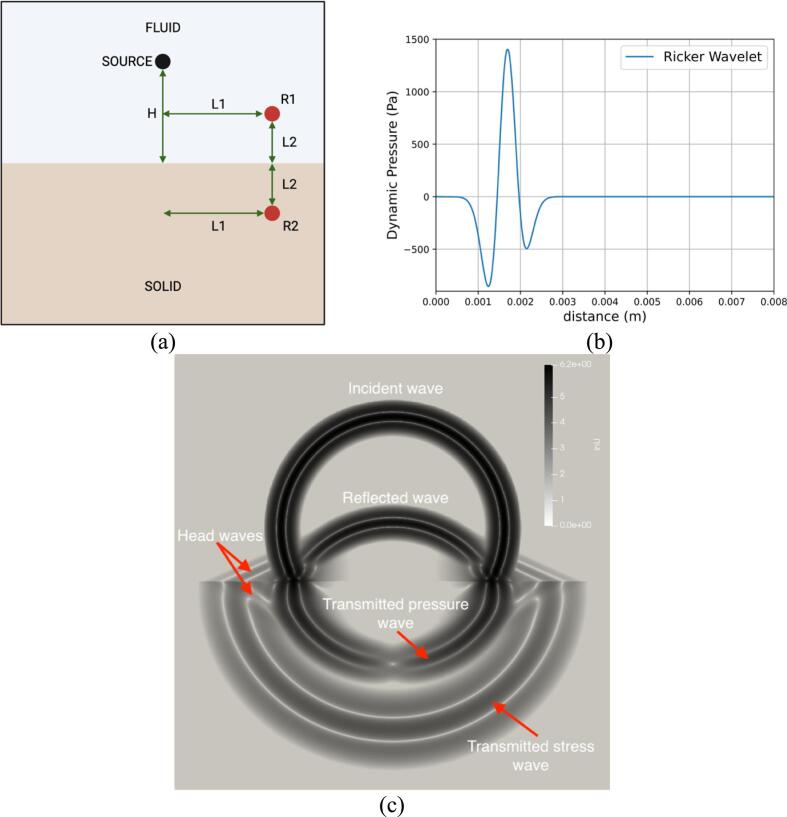
(a) Wave transmission across fluid/solid interface schematic. (b) Pressure profile of the Ricker wavelet generated from the monopole source. (c) Velocity magnitude at t = 3μs. The incident and reflected waves in the fluid, the transmitted stress and pressure waves as well as head waves in both materials are clearly captured.

A semi-analytical wave propagation case is presented as shown in Fig. 2a. A 3D domain is utilized where the upper half is the fluid material, and the lower half is solid. In the fluid medium, a spherical pressure wave is generated from a monopole source, similar to a spherical shock emitted by a collapsing bubble. The monopole source is standing at a distance H from the planar fluid/solid interface. To validate the results between the semi-analytical solution and the numerical results, two sensors $R_{1}$, and $R_{2}$ were placed in the fluid and solid medium, respectively. The sensors were placed symmetrically at a distance $L_{1}=2.5mm$ from the axis of symmetry and at a distance $L_{2}=1.45mm$ above and below the planar interface. The dynamic pressure was recorded at the sensor inside the fluid medium. Additionally, the horizontal and vertical velocities were recorded inside the solid medium.

The Ricker wavelet was chosen for the spherical pressure wave. The wavelet was prescribed as an initial condition in the fluid medium where the pressure at a distance r from the source is expressed as:

(26) $$p\left(r\right)=p_{0}+\frac{Q(-r/c_{0})}{r}$$

(27) $$Q\left(s\right)=\left(1-2\pi^{2}f_{0}^{2}{\left(s+s_{1}\right)}^{2}\right)e^{-\pi^{2}f_{0}^{2}{\left(s+s_{1}\right)}^{2}}$$

with $p_{0}$ the hydrostatic pressure, $c_{0}$ the speed of sound of the fluid medium, $f_{0}$ is the frequency of the wavelet and $s_{1}$ is a constant that controls the initial position of the wavelet. The parameters used are given in Table 1.

**Table 1:** Thermodynamic and wavelet parameters of the semi-analytical wave transmission problem.

Ricker wavelet		Fluid		Solid	
$f_{0}$(MHz)	1.43	$\rho_{f}$(kg/$m^{3}$)	1000	$\rho_{s}$(kg/$m^{3}$)	1995
$s_{1}$(μs)	0.85	$\gamma_{f}$	6.59	$\gamma_{s}$	3.4
H(mm)	2.9	$p_{\infty ,f}$(MPa)	410	$p_{\infty ,s}$(Pa)	4,591,545,000
$p_{0}$(MPa)	0.101	$\mu_{f}$(Pa)	0	$\mu_{s}$(Pa)	10,728,633,195

In the presented problem, the maximum dynamic pressure within the fluid is two orders of magnitude less than the hydrostatic pressure. Thus, we can interpret the impacting wave as a small disturbance, and therefore this can be modeled as an acoustic wave propagating through a homogenous fluid medium. Under the assumption of linear elasticity and isotropy of the solid material, the overall problem can be solved analytically by coupling the linear acoustic wave equation with the equation of motion for a linear elastic solid. Such an analytical solution can be obtained with the Cagniard-de Hoop method [82]. A semi-analytical solution was computed using the open-source software, Gar6more3D [83].

**Fig. 3:**
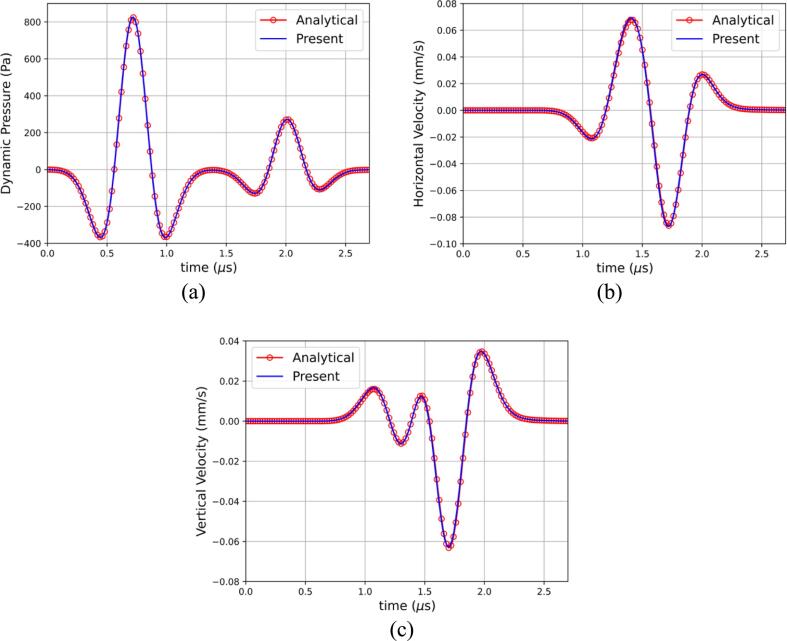
Comparison between the present numerical results and the semi-analytical results of Gar6more3D for (a) the dynamic pressure, (b) horizontal velocity, and (c) vertical velocity.

The simulation was carried out in axisymmetric coordinates with β=1 as detailed in Eq (18) to save computational time. The computational domains span 8 mm in the axial direction and 16 mm in the vertical direction. A symmetry boundary condition was imposed at the center axis and outflow boundary conditions at all the other boundaries. The base mesh consists of 256 cells in the axial direction and 512 cells in the vertical direction. The adaptive mesh refinement was allowed to refine up to 2 levels according to the magnitude of the density gradient. A CFL number of 0.4 was used to compute the timestep. The thermodynamic parameters for the simulation can be found in Table 1. The velocity magnitude is shown in Fig. 2a where the incident and reflected waves in the fluid, the transmitted stress and pressure waves as well as head waves in both materials are clearly captured. The numerical results are compared to the semi-analytical solution of Gar6more3D in Fig. 3 where an excellent agreement is found.

### 4.3 Ultrasound bubble rigid wall interaction

**Fig. 4:**
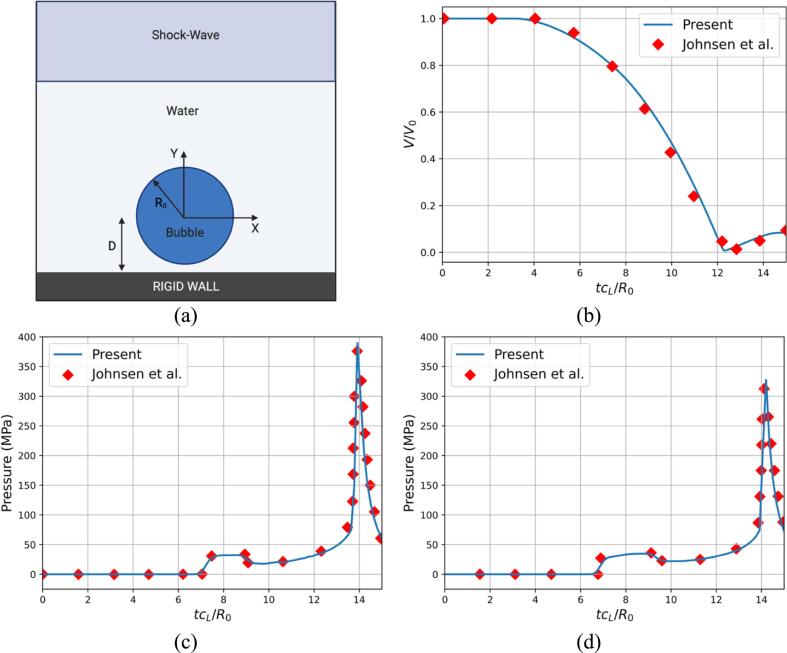
The ultrasound-induced collapse of an air bubble near a rigid wall at a standoff distance $S_{d}=2.0\;R_{0}$. Comparison of the present results with the reference [84]. (a) Schematic of the ultrasound-bubble-rigid wall problem (b) Temporal evolution of the normalized air volume (c) Temporal evolution of the pressure at probe location ${x/R}_{0}=0$ (d) Temporal evolution of the pressure at probe location ${x/R}_{0}=1$.

The ultrasound-bubble rigid wall interaction is validated against published results [84]. The case features a 35 MPa lithotripter pulse impacting an air bubble of initial radius $R_{0}$ above a rigid wall at a distance D. The schematic of the case is shown in Fig. 4a. The computational domain spans ${L_{x}=5R}_{0}$ and ${L_{y}=10R}_{0}$ where $R_{0}=50\mu m$. To save computational time, the simulations were conducted using an axisymmetric formulation with β=1 as detailed in Eq (18). The base mesh of $N_{x}=256$ and $N_{y}=512$ cells is used, resulting in 131,072 initial cells prior to refinement; the corresponding mesh spacing is $\Delta x=9.76\cdot 10^{-4}\;mm$. Two levels of refinement have been used. The top boundary is used to propagate the lithotripter pulse while an outflow boundary condition is imposed on the right; the left boundary is the axis of symmetry, and the bottom boundary is a slip wall. A CFL number of 0.1 was imposed to preserve the explicit scheme's numerical stability. The density of the water is initially $\rho_{\mathrm{water}}=998.2kg\cdot m^{-3}$ while the thermodynamic parameters for the water are $\gamma_{\mathrm{water}}=6.59$, $p_{\infty ,water}=4049atm$ as reported in [84]. The parameters for the air are $\gamma_{\mathrm{air}}=1.4$, $p_{\infty ,air}=0$ which results in considering an ideal gas with an initial density $\rho_{\mathrm{air}}=1.225kg.m^{-3}$.

The comparison between the present results and the reference is presented in Fig. 4b-d. The temporal evolution of the dimensionless air volume and the wall pressure at two probe locations are shown. The collapse of the bubble is initiated by the ultrasound impact leading to an adverse pressure gradient forming. The pressure elevation due to the incoming ultrasound is seen in Fig. 4c-d as the first spike in pressure. The following decrease in pressure is the rarefaction wave coming from the bubble interface. The ultrasound is reflected off the rigid wall and impacts the bubble a second time precipitating the collapse. The emitted spherical shock by the bubble collapse is captured by both probes. The results are found to be in excellent agreement with the reference [84] as seen in Fig. 4a-d.

## 5 Results and discussion

**Fig. 5:**
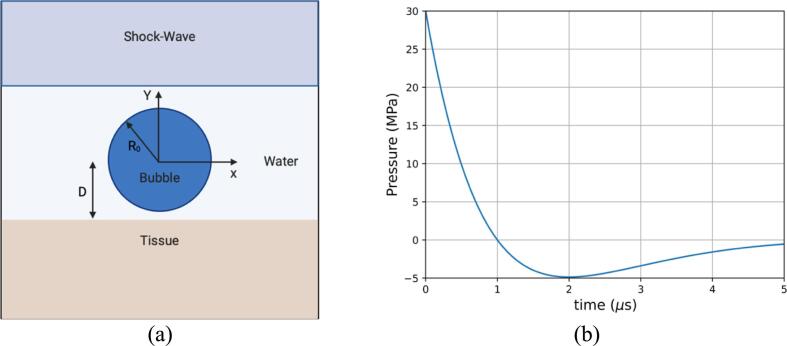
(a) Schematic of the ultrasound-bubble-tissue problem (b) Temporal evolution of the lithotripter pulse.

The objective of this section is to provide a deeper understanding of the complex dynamics of the ultrasound-induced collapse of bubbles near soft materials. More specifically, the case of an ultrasound-induced bubble collapse near tissue-mimicking materials has been simulated; an in-depth explanation of the first case simulated is initially presented, followed by cases revealing the effects of shear modulus, bubble radius, and the standoff distance on the deformation and stresses developed in the deforming soft material. The gas bubble is at mechanical equilibrium with the surrounding medium initially. The schematic of the case is presented in Fig. 5a:

The ultrasound shockwave is chosen to be similar to that produced by a Dornier HM3 lithotripter where $p_{s}=30MPa$ is the pulse amplitude, $\alpha =9.1\cdot 10^{5}$ and ω=2πf with $f=83.3\cdot 10^{3}Hz$. The analytical function of the waveform [85] is defined as:

(28) $$p\left(t\right)=p_{0}+2p_{s}e^{-\alpha t}\cos\left(\omega t+\frac{\pi }{3}\right)$$

where $p_{0}$ is the atmospheric pressure. The temporal evolution of the lithotripter pulse is shown in Fig. 5b. The bubble radii under consideration were chosen to reflect typical bubble sizes in diagnostic and therapeutic applications of bubbles in medicine [1], [86] where $R_{0}=10\mu m$, $R_{0}=5\mu m$, $R_{0}=2.5\mu m$. Three soft materials have been considered; their shear modulus spans over 3 orders of magnitude, which correspond to three well-characterized tissues [87]: liver, gallbladder, and bile duct tissue [88]. The corresponding parameters of the EoS utilized to characterize them are summarized in Table 2.

**Table 2:** Thermodynamics parameters of the three soft materials.

Tissue	ρ($kg.m^{-3}$)	μ(Pa)	γ	$p_{\infty }$(Pa)
Liver	1060	$1,8\cdot 10^{3}$	4.4	599023259
Gallbladder	1060	$8,5\cdot 10^{4}$	4.4	616576970
Bile duct	1060	$1,66\cdot 10^{5}$	4.4	604328642

To save computational time, the simulations were conducted using an axisymmetric formulation with β=1 as detailed in Eq (18). The computational domain spans at $L_{x}=0.25$ mm and $L_{y}=0.5mm$. The base mesh of $N_{x}=256$ and $N_{y}=512$ cells is used, resulting in 131,072 initial cells prior to refinement; the corresponding mesh spacing is $\Delta x=9.76\cdot 10^{-4}\;mm$. Three levels of refinement have been considered, based on the refinement criterion. The top boundary is used to propagate the lithotripter pulse ( 2 8 ) while an outflow boundary condition is imposed on the right and lower boundaries; the left boundary is the axis of symmetry. To preserve the numerical stability of the explicit scheme, a CFL number of 0.1 was imposed in all subsequent simulations.

With regard to the interaction between the collapsing bubbles and the nearby solid material, many studies have been reported [46], [48], [89], [90], [91], [92]. Cavitation damage in solid materials primarily stems from shock waves and liquid jets produced during the bubble's collapse. The shock waves exert pressure spikes that can lead to fatigue and micro-cracking, while the liquid jet's impact can cause pitting and erosion; repeated occurrences can lead to severe degradation of the surface. For soft materials, it is generally known that the liquid jet is the prevailing mechanism for their damage [92], [93]. Biological tissue can be damaged through various mechanical loads, including compressive, tensile, and shear forces [94]. These forces can lead to different types of damage or injury depending on the nature and duration of the applied force, and the type of tissue [95]. The type of tissue (e.g., bone, muscle, ligament) and the specific mechanical properties of that tissue will determine its susceptibility to different types of mechanical forces. For example, bones [96] are more resilient to compressive forces but can be more vulnerable to tensile and torsional forces. Soft tissues which are considered in this paper, like muscles and ligaments might be more prone to damage through excessive stretching or tensile forces [97]. The specific damage mechanism of bubble collapse for hard materials such as metals is widely debated [79]. To further elaborate on the details of this mechanism, the maximum, minimum principal stress and maximum shear stress are computed:

(29) $$\sigma_{\max,PS}=\frac{\sigma_{\mathrm{xx}}+\sigma_{\mathrm{yy}}}{2}+\sqrt{{\left(\frac{\sigma_{\mathrm{xx}}-\sigma_{\mathrm{yy}}}{2}\right)}^{2}+{\sigma_{\mathrm{xy}}}^{2}}$$

(30) $$\sigma_{\min,PS}=\frac{\sigma_{\mathrm{xx}}+\sigma_{\mathrm{yy}}}{2}-\sqrt{{\left(\frac{\sigma_{\mathrm{xx}}-\sigma_{\mathrm{yy}}}{2}\right)}^{2}+{\sigma_{\mathrm{xy}}}^{2}}$$

(31) $$\sigma_{\mathrm{MSS}}=\sqrt{{\left(\frac{\sigma_{\mathrm{xx}}-\sigma_{\mathrm{yy}}}{2}\right)}^{2}+{\sigma_{\mathrm{xy}}}^{2}}$$

By visualizing these stresses, the directional nature of the stress state in the material and areas of potential material failure can be understood and identified. Particularly, areas, where the maximum principal stress (tensile) exceeds the tensile strength of the material, are likely failure points.

### 5.1 Dynamics of the ultrasound-bubble tissue interaction

**Fig. 6:**
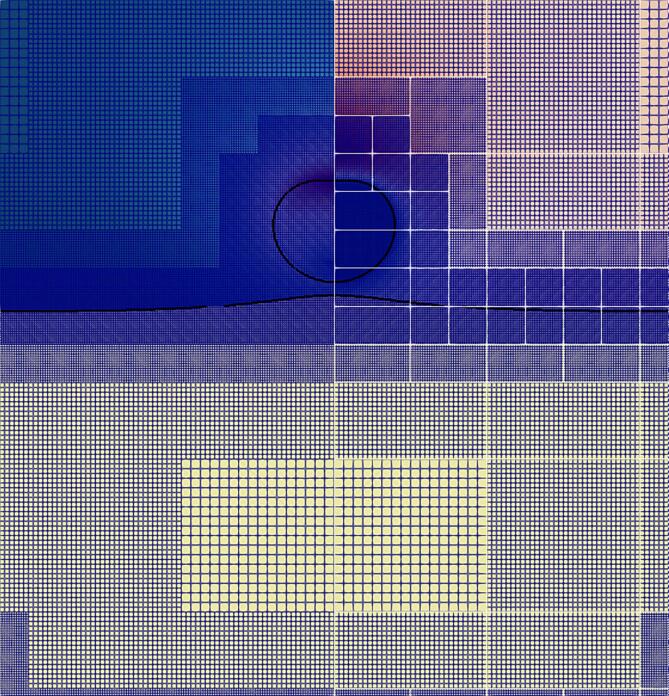
Block-structured adaptive mesh refinement visualization of the ultrasound-induced collapse of an air bubble of $R_{0}=10\mathrm{\mu m}$ with an initial standoff $S_{d}=1.1R_{0}$ near gallbladder. The white outline delimits the blocks.

In this section we present an in-depth analysis of the ultrasound-induced collapse of an air bubble with a radius $R_{0}=10\mu m$ placed above a soft material at a standoff distance $S_{d}=1.1R_{0}$ whose properties are representative of the gallbladder. The corresponding parameters of the EoS utilized to characterize it are summarized in Table 2. In Fig. 6, the block-structured adaptive mesh refinement is depicted.

**Fig. 7:**
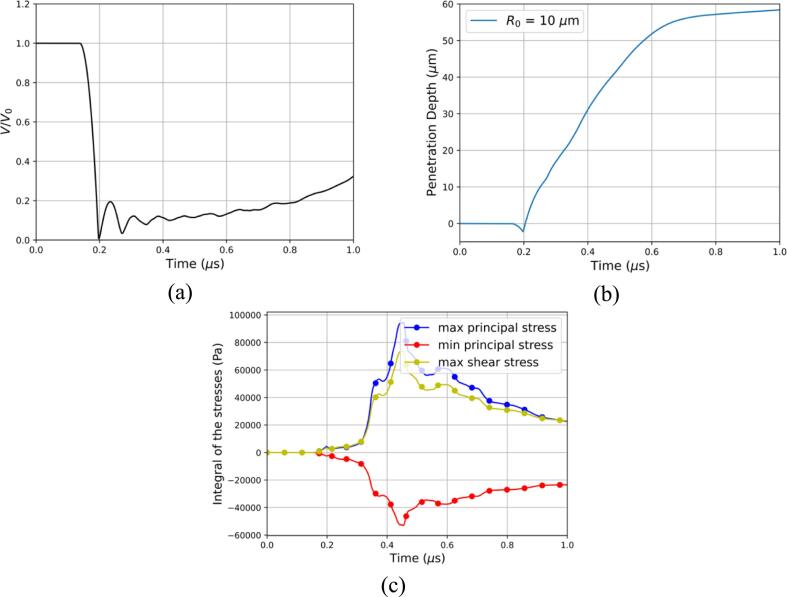
Ultrasound-induced collapse of an air bubble of $R_{0}=10\mathrm{\mu m}$ with an initial standoff $S_{d}\;=\;1.1\;R_{0}$ near gallbladder (a) Temporal evolution of the normalized air volume (b) Temporal evolution of the penetration depth of the liquid jet (c) Temporal evolution of the integral of the maximum, minimum principal stress, and maximum shear stress.

**Fig. 8:**
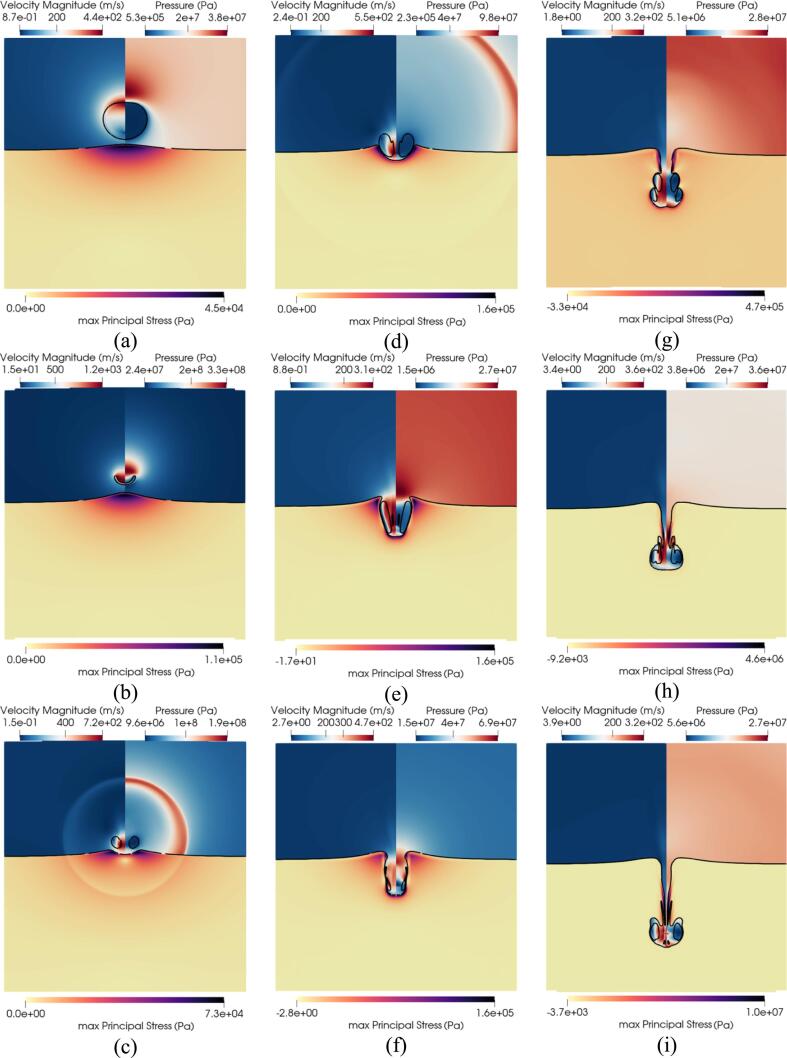
Ultrasound-induced collapse of an air bubble of $R_{0}=10\mathrm{\mu m}$ with an initial standoff $S_{d}\;=\;1.1\;R_{0}$ near gallbladder at different timesteps. The black isosurface separates the three materials and is defined by $\alpha_{k}=0.5.$ Upper left hand: contour of velocity magnitude. Upper right hand: contour of pressure. Bottom half: contour of the maximum principal stress.

The temporal evolution of the air volume normalized by its initial value is presented in Fig. 7a. The bubble remains at its initial radius until the shockwave impacts it leading to the generation of a pressure gradient. The temporal evolution of the penetration depth into the tissue at the center axis can also be seen in Fig. 7b. From Fig. 7b we can identify 3 different stages: the collapse stage up to 0.2μs, followed by the penetration stage until 0.6μs, and finally the tissue rebound stage where the penetration of the liquid jet slows down due to elastic forces. The collapse process of the bubble starts 0.15μs, and it reaches its minimum radius of around 0.2μs. The tissue is pulled upwards during the first collapse as evidenced by the negative penetration depth in Fig. 7b. This is also shown in Fig. 8b. The temporal evolution of the surface integrals of the maximum, minimum principal stress, and maximum shear stress over a small area of the tissue were computed and shown in Fig. 7c. We observe a slight increase in principal and shear stress at collapse suggesting the emitted shockwave produced a small deformation. Given the small acoustic impedance mismatch between the water and the tissue, here modeled as gallbladder the stresses produced are rather insignificant. After the first collapse, the liquid jet starts penetrating the tissue where multiple bubble collapses and rebounds are observed Fig. 7a. It is during the penetration stage that the tissue experiences the highest stresses as depicted in Fig. 7c. The maximum principal stress is observed to be approximately double the minimum principal stress around 0.42μs showing that the tensile forces are much greater than the compressive forces. This is an important finding as soft materials like biological tissue are more susceptible to tensile damage as explained in section 5. In the last stage of this process, the bubble experiences smaller collapses and rebounds and on average increases in volume Fig. 7a. However, the penetration of the liquid jet has been slowed as can be seen by the decrease in slope in Fig. 7b after 0.6μs.

In Fig. 8, the ultrasound-induced collapse near gallbladder is depicted for nine different timesteps with a black isosurface separating the three materials. The maximum principal stress inside the tissue, the velocity magnitude and pressure in the water and air are depicted. In the first instance Fig. 8a, the lithotripter pulse already impacted the bubble and propagated inside the tissue. Given the very similar acoustic impedance between water and gallbladder, the pulse is entirely transmitted inside the tissue. At the distal side of the bubble a rarefaction wave propagating outward was generated given the impedance mismatch between the water and air phase.

The dynamic of bubbles during extracorporeal shockwave lithotripsy near gelatin was studied in [98] using a 10.2 MPa pulse and millimeter sized bubbles. Both the transmission of the lithotripter pulse into the gelatin and the rarefaction wave have been experimentally observed.

In Fig. 8a, the adverse pressure gradient is seen where the distal side of the bubble contracted starting the collapse process. The low-pressure region at the proximal side of the bubble creates a sink flow where the tissue is pulled toward the bubble.

Although the elongation of the tissue towards the bubble during the first collapse has not been observed in [98], it has been documented in laser-induced bubbles close to tissue mimicking materials [56], [99], [100]. The limitations in movie resolutions of the experimental setup could be responsible. Moreover, the weaker lithotripter pulse and a bigger bubble size in their experiment contributes to a weaker collapse, and thus resulting in a weaker sink flow.

The deformation induces stresses in a spherical like shape where the stresses are highest right under the bubble and gradually decreases to zero further away. Moments before the collapse in Fig. 10b, the liquid jet peaks at velocities 1200m/s and the tissue is seen to have been further pulled inwards towards the bubble where the maximum principal stress has increased by an order of magnitude. The high velocity liquid jet has been reported in experiments to be able to perforate membranes like aluminum foil [101].

**Fig. 9:**
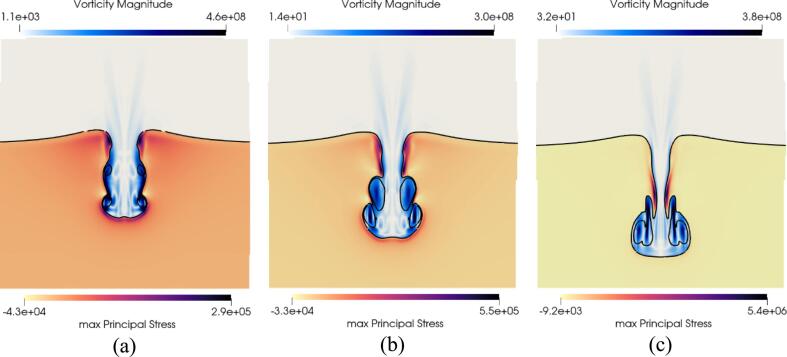
Contours of vorticity and maximum principal stress at different timesteps.

**Fig. 10:**
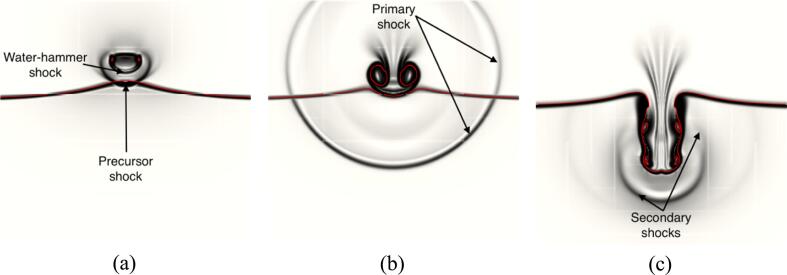
Numerical schlieren at different timesteps where the red isosurface represents the interface between the three materials. (For interpretation of the references to colour in this figure legend, the reader is referred to the web version of this article.)

To better visualize the shock waves numerical schlieren were plotted in Fig. 10 based on the following formula [102]:

(32) $$\phi =\exp\left(-\frac{k\left|\nabla \rho \right|}{max\left|\nabla \rho \right|}\right)$$

In Fig. 10a, the water-hammer shock, and the precursor shock right after the collapse are seen. Once the liquid jet penetrates the proximal side of the bubble a toroidal bubble is observed in Fig. 8c. The subsequent collapse of the bubble generates a spherical shock wave traveling outward both in the water and the tissue where small stresses are observed. This primary shock is also shown in Fig. 10b. The liquid jet already pushed the tissue inward, compressing it in Fig. 8c and started to penetrate. The shape of the maximum principal stress has changed compared to the pre-collapse phase. The highest maximum principal stress at this timestep is observed in the periphery of the impact location while compressive forces are seen at the centroid. The liquid jet continues its inward motion inside the tissue as depicted in Fig. 8d while the bubble rebounds and increases in size. Both the bubble and the liquid jet are then fully surrounded by the tissue in Fig. 8e where the bubble is seen with an elongated shape. A second smaller toroidal bubble is also observed. The upper part of the tissue forming a tip and the impact location is seen to experience significant tensile forces while the sides are seen to be compressed.

At this point, the toroidal bubble collapses for the second time as seen in Fig. 8f, and an upward moving shock is emitted.

In [98] the shockwave-induced collapse of a bubble near gelatin is described. The shock due to bubble collapse is first seen, followed by the liquid jet impacting the gelatin and compressing it similarly as in Fig. 8c. The maximum principal stress in Fig. 8c shows an inflection point where the tissue is not only compressed at the impact point but also experiences tensile forces when moving away from the center. The bubble is then observed in [98] to both penetrate the gelatin and rebound while the centroid of the bubble is moving downstream which is consistent with Fig. 8d. Notably, the primary shock depicted in Fig. 9b and a secondary shock is observed in [98] although in their experiments it is not clear if the bubble splits into two toroidal bubbles since it is not possible to observe inside the gelatin.

The tip of the tissue is also observed to be retracting downwards while the shape of the stresses remains similar to Fig. 8e. In Fig. 9, the vorticity magnitude in the two fluid phases and the maximum principal stress in the tissue are shown. High vorticity regions are observed along the outward tissue walls moments after the secondary collapse as seen in Fig. 9a. This vorticity is responsible for deforming the tissue interface that was previously planar into a curved shape Fig. 9b. The subsequent secondary collapse created 2 toroidal bubbles that rebounded as seen in Fig. 8g where the tissue is pinched between the two bubbles. The upper part is getting stretched downward and hence experiences tensile forces close to the water-tissue interface. The secondary collapse shocks are both shown in Fig. 10c. As the liquid jet continues its downward motion, the two toroidal bubbles experience a third collapse; significant vorticity is present, as shown in Fig. 9c. Finally, the two bubbles merged into one once again, as shown in Fig. 8h. The part of the tissue above the upstream torus is pulled downward forming a spike, where most of the tensile forces are present. As the penetration process inside the tissue continues, the spike is further pulled downstream, as evidenced in Fig. 8i. The maximum principal stress is notably highest very close to the interface where the tissue was penetrated and particularly in the spikes.

In various experimental studies, the aftermath of bubble collapse is characterized by the formation of a pit within the soft material, serving as evidence of material damage [10], [56], [98], [99], [103]. The primary mechanism responsible for such damage is seemingly attributed to the liquid jet. The present results corroborate these findings and elucidate the dynamic processes that lead to tissue damage. Moreover, the probable sites of such damage are identified, enhancing our understanding of the damage locations within the tissue.

### 5.2 Effect of the shear modulus

**Fig. 11:**
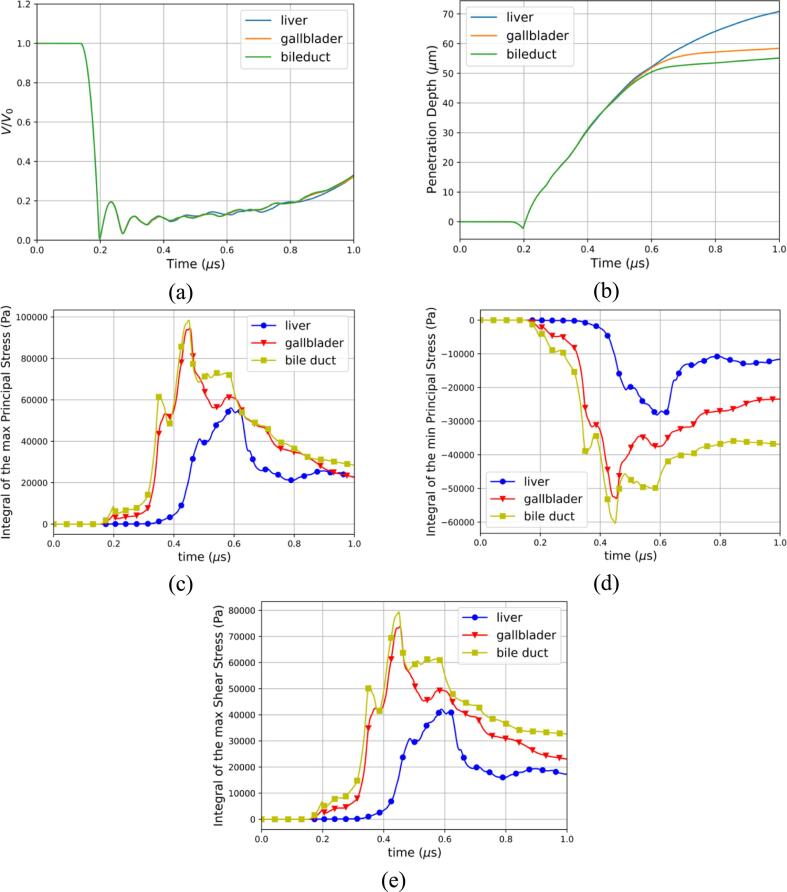
Effect of the shear modulus (a) Temporal evolution of the normalized air volume (b) Temporal evolution of the penetration depth of the liquid jet for 3 different tissues (c) Integral of the maximum principal stress for 3 different tissues (d) Integral of the minimum principal stress for 3 different tissues (e) Integral of the maximum shear stress for 3 different tissues.

**Fig. 12:**
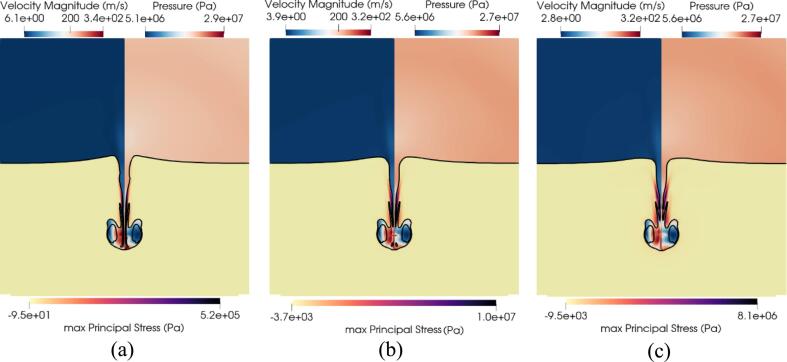
Effect of the shear modulus on the shape of the deformation at t=0.373μs (a) Tri-contours of the liver (b) Tri-contours of the gallbladder (c) Tri-contours of the bile duct.

In this section, the effect of the shear modulus on both the bubble and solid dynamics was studied by selecting two additional tissues: liver and bile duct. The corresponding parameters of the EoS utilized to characterize them are summarized in Table 2. In Fig. 11, the temporal evolution of the normalized air volume, penetration depth and surface integrals of the stresses are presented. The change in shear modulus is seen in Fig. 11a to have a small impact on the bubble dynamics. The early stage of the bubble dynamics is observed to be identical up to the third collapse where the normalized air volume starts to vary for the different tissues. This finding is consistent with the hypothesis that the early stage of the simulation is ultrasound-driven due to the small acoustic impedance mismatch. In Fig. 11b, the penetration depth into the tissues is observed to be similar up to the end of the penetration stage. Later the speed of the penetration slows down and the tissue with the lowest shear modulus, the liver is clearly seen to be penetrated more than the strongest soft tissue selected: the bile duct. The surface integrals of the maximum, minimum principal stress and maximum shear stress are plotted in Fig. 11c-e. The increase in shear modulus is observed to induce higher stresses across the 3 metrics measured. It is consistent with the fact that the strongest soft tissue deforms less than the softest and can also be seen in Fig. 11b with the penetration depth. The effect of the shear modulus on the penetration depth and deformation has also been assessed in experiments [103]. It was observed that both the penetration depth and the deformation of the tissue-mimicking material decreased with increasing shear modulus. The same observations are made in this work whereby the increase of the shear modulus is demonstrated to decrease the penetration depth in late stages as depicted in Fig. 11b, and the deformation seen by the reduction in elongation in Fig. 12. Across the 3 metrics of stress measured and for the selected tissues, the maximum principal stress is seen to be the highest.

In Fig. 12, the tri-contours for the liver, gallbladder, and bile duct respectively at t=0.373μs are presented. While the shape of the deformation overall is similar, there are two noticeable differences. At this stage of the process, the upstream entry point for the three different tissues is seen to have different shapes. Indeed, the bile duct which is the strongest soft tissue is observed to have retracted faster than the gallbladder and the liver respectively. Additionally, the entrainment of the tissue due to the vorticity is less prominent with the increase in shear modulus. It can be seen in the three figures where the elongated tissue for the liver reaches downstream close to the liquid jet pit. Whereas in the case of the gallbladder the elongated tissue is above the toroidal bubble at the same timestep. Similarly, in the case of the bile duct the elongation of the tissue is greatly reduced compared to the two other tissues at that time. Lastly, the bubble shape, velocity and pressure are observed to be the same in the three cases presented demonstrating again that the increase in shear modulus does not impact the bubble dynamics at this stage of the process.

### 5.3 Effect of the bubble radius

**Fig. 13:**
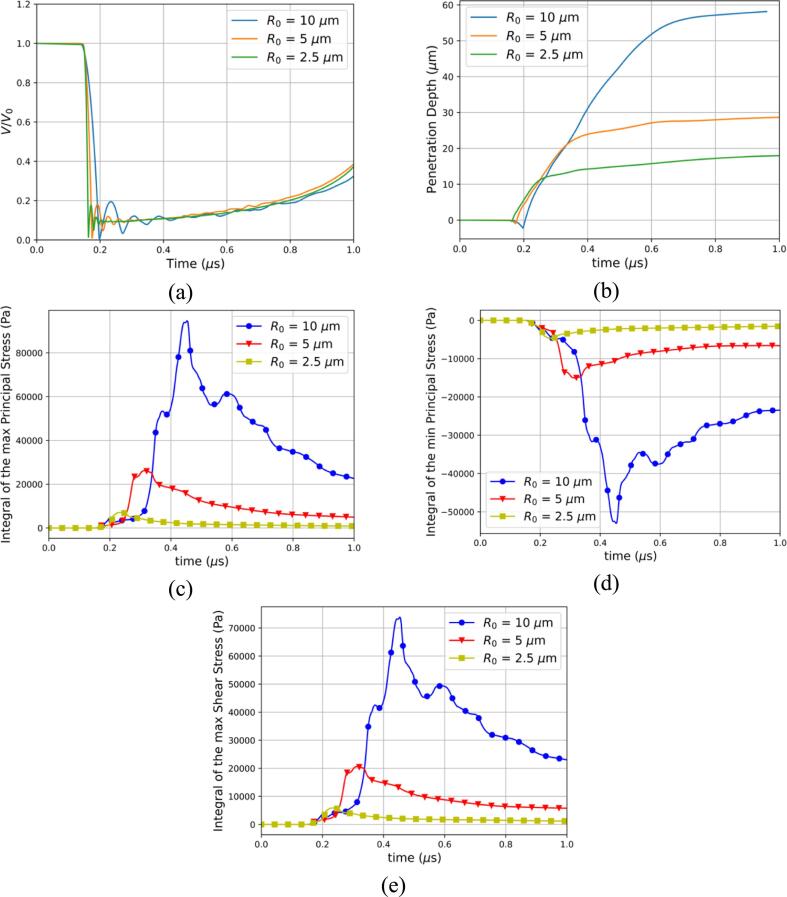
Effect of the initial bubble radius (a) Temporal evolution of the normalized air volume (b) Temporal evolution of the penetration depth of the liquid jet for 3 different bubble radii (c) Integral of the maximum principal stress for 3 different bubble radii (d) Integral of the minimum principal stress for 3 different bubble radii (e) Integral of the maximum shear stress for 3 different bubble radii.

In this section, the effect of the bubble radius is investigated by selecting bubble radii found in therapeutic applications [86]. The initial bubble radii $R_{0}$ are chosen to be 10μm, 5μm, and 2.5μm and gallbladder is selected for the three cases presented. In Fig. 13, the temporal evolution of the normalized air volume, penetration depth and surface integrals of the stresses are presented for the different bubble radii. First, the bubble dynamics are naturally seen to be significantly different in Fig. 13a where the collapse times, minimum radii, and rebound radii change. Second, the bubble radius is seen to have a major effect on the penetration depth in Fig. 13b. Indeed, all three identified stages are affected. In the collapse stage, the pulling effect of the tissue is less prominent for the smaller bubbles. This result is consistent with our hypothesis that the main mechanism behind the pulling effect is the sink flow below the bubble. The penetration stage is observed to be shortened for the smaller bubbles. It can be attributed to the fact that the collapse is milder as the bubble is smaller and the subsequent liquid jet reaches smaller velocities. The rebound stage although happening earlier exhibits the same behavior for all radii where the penetration of the velocity liquid jet is greatly reduced. Lastly, another major finding is the importance of the bubble size on the stresses experienced by the tissue. In Fig. 13c-e, the maximum, minimum principal stresses, and maximum shear stress are plotted. The trend of the bigger bubble to produce higher stresses is clearly observed.

The effect of the initial bubble radius on the deformation and the penetration depth was examined in [98], [103]. It was found that larger bubble resulted in an increased penetration depth as well as an increase in the damage pit radius. In Fig. 13b, the same correlation of the penetration depth on the initial bubble radius is found. This finding has significant importance for mitigating cavitation-related injuries where bubble sizes can be carefully chosen for specific treatments.

### 5.4 Effect of the standoff distance

**Fig. 14:**
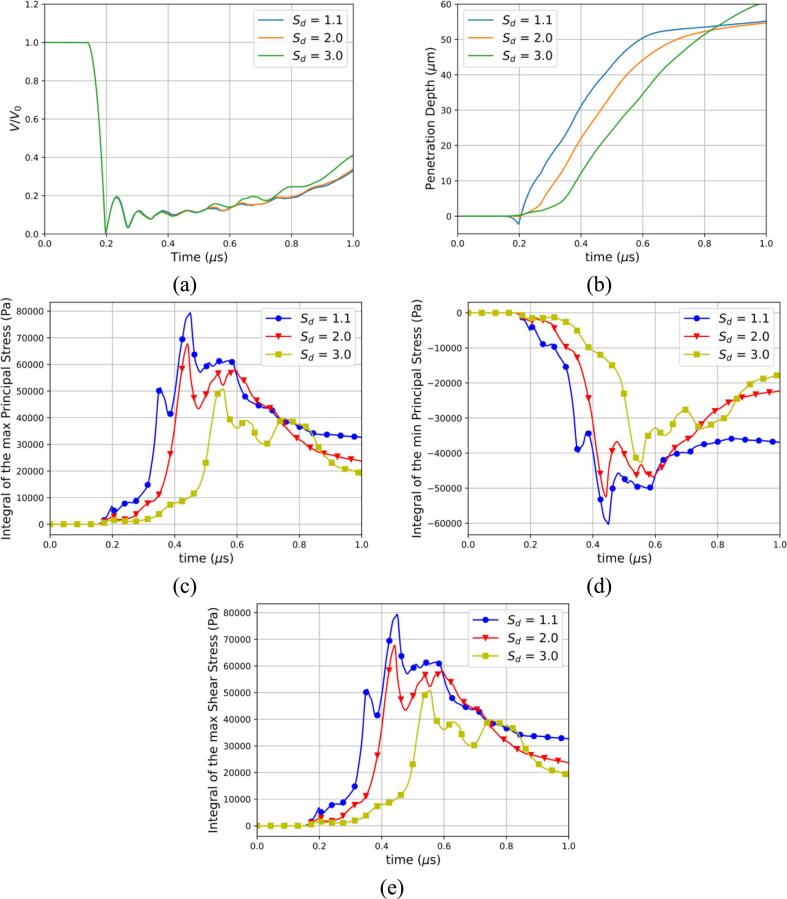
Effect of the standoff distance (a) Temporal evolution of the normalized air volume (b) Temporal evolution of the penetration depth of the liquid jet for 3 different standoff distances (c) Integral of the maximum principal stress for 3 different standoff distances (d) Integral of the minimum principal stress for 3 different standoff distances (e) Integral of the maximum shear stress for 3 different standoff distances.

In this section, the effect of the standoff distance on the bubble dynamics and the deformation is presented herein. Three standoff distances are examined: $S_{d}=1.1R_{0}$, $S_{d}=2.0R_{0}$, $S_{d}=3.0R_{0}$ where $R_{0}=10\mathrm{\mu m}$. The temporal evolution of the air volume normalized by its initial value is depicted in Fig. 14a for the three standoff distances. The bubble’s collapse time is observed to be the same for all three cases. Differences in bubble dynamics become more pronounced after the third collapse, with the greatest standoff distance showing the most significant variations. For two smallest standoff distances the second and third collapse occur while the bubble is engulfed by the tissue which is not the case for $S_{d}=3.0R_{0}$. We observed that this difference could be due to the elastic forces the bubble must overcome to grow while engulfed in the tissue. The temporal evolution of the penetration depth into the tissue at the center axis can also be seen in Fig. 14b for the three standoff distances. The suction effect during which the tissue is pulled upstream towards the bubble for $S_{d}=1.1R_{0}$ is not observed for $S_{d}=2.0R_{0}$ and $S_{d}=3.0R_{0}$. The velocity of the penetration depth as depicted by the slope in Fig. 14b is seen to be similar during the penetration process for all standoff distances. The later dynamics exhibit a notable difference where the two smallest standoff distances reach the tissue rebound stage sooner than the largest standoff distance. The temporal evolution of the surface integrals of the maximum, minimum principal stress and maximum shear stress are presented in Fig. 14c-e. A clear correlation between the standoff distance and the stresses is observed. The smallest standoff distances are depicted to induce higher stresses. These insights are pivotal for the development of strategies aimed at mitigating cavitation-induced tissue damage in therapeutic applications.

**Fig. 15:**
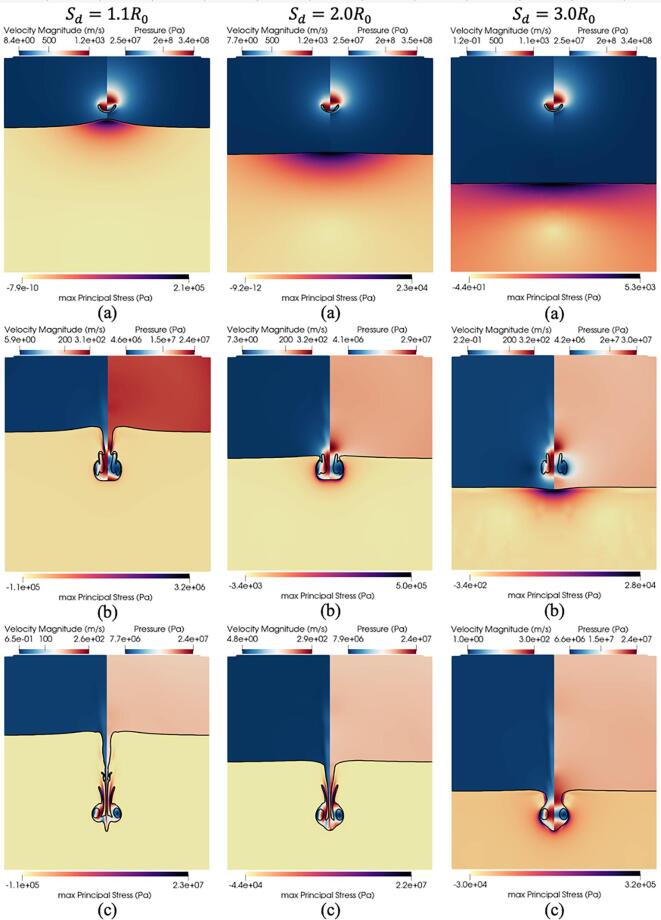
Effect of the standoff distance. Ultrasound-induced collapse of an air bubble of $R_{0}=10\mathrm{\mu m}$ near bileduct at t=0.19μs, t=0.31μs, and t=0.44μs for standoff distances $S_{d}=1.1R_{0}$, $S_{d}=2.0R_{0}$ and $S_{d}=3.0R_{0}$. The black isosurface separates the three materials and is defined by $\alpha_{k}=0.5.$ Upper left hand: contour of velocity magnitude. Upper right hand: contour of pressure. Bottom half: contour of the maximum principal stress.

In Fig. 15, the effect of the standoff distance on the ultrasound-induced collapse of an air bubble of $R_{0}=10\mu m$ near bileduct for $S_{d}=1.1R_{0}$, $S_{d}=2.0R_{0}$ and $S_{d}=3.0R_{0}$ is depicted for three timestep at t=0.19μs, t=0.31μs, and t=0.44μs. The first presented timestep at t=0.19μs in Fig. 15a demonstrates the effect of the standoff distance on the suction effect. As the standoff distance increases the protrusion of the tissue upstream toward the bubble decreases. This trend corroborates the proposed hypothesis, implicating the sink flow beneath the bubble as the primary mechanism driving this protrusion. The second presented timestep at t=0.31μs in Fig. 15b corresponds to the third collapse as seen in Fig. 14a. The early bubble dynamics are shown not to be affected by the standoff distance as the shape of the bubble remains the same across the three standoff distances. Nevertheless, the bubble exhibits distinct phases within the penetration process, as evidenced by the observed variations in penetration depth in Fig. 15b. In fact, the lowest standoff distance $S_{d}=1.1R_{0}$ is seen to experience the highest stresses at this timestep as evidenced in Fig. 14c-e. The observations at the final presented timestep at t=0.44μs in Fig. 15c are consistent with earlier findings, showing that the bubble maintains its shape across the various standoff, yet is situated at different penetration depths and experiences higher stresses as the standoff distance decreases.

## 6 Conclusion

A numerical investigation of the ultrasound-induced collapse of air bubbles near soft materials was presented using a novel multi-material DIM model with AMR. To have a better understanding of the complex interactions of the ultrasound-bubble-tissue flow, the effect of the shear modulus, the bubble radius and the standoff distance were investigated. The shear moduli were chosen for well-characterized tissues spanning over three orders of magnitude. The bubble radii considered are found in biological flows. Insights into the nature of the mechanical loads experienced by the soft material through the visualization of the maximum and minimum principal stress and maximum shear stress were presented.

Our findings reveal that the tissue predominantly experiences tensile forces compared to compressive or shear forces, suggesting that injuries are mainly tensile-driven. Concurrently, areas of maximum tensile forces align closely with regions where the tissue undergoes elongation. Furthermore, the bubble radius is identified to play a pivotal role in the stresses experienced by the soft material, emphasizing its importance in medical applications. Meanwhile, variations in shear modulus, while having a minimal impact on early bubble dynamics, noticeably influence the penetration process in later stages as well as the shape of the deformations. Finally, it is found that smaller standoff distances lead to greater bubble-tissue interaction resulting in higher stresses in the tissue while the bubble dynamics are not notably affected.

This work contributes valuable insights into the complex interplay between bubble collapse, acoustic fields, and tissues, paving the way for improvements in related medical applications.

## CRediT authorship contribution statement

**Armand Shams:** Conceptualization, Data curation, Formal analysis, Investigation, Methodology, Software, Validation, Visualization, Writing – original dravft, Writing – review & editing. **Saeed Bidi:** Conceptualization, Methodology, Writing – review & editing. **Manolis Gavaises:** Conceptualization, Funding acquisition, Project administration, Resources, Supervision, Writing – review & editing.

## Declaration of competing interest

The authors declare that they have no known competing financial interests or personal relationships that could have appeared to influence the work reported in this paper.

## Figures and Tables

**Fig. 16:**
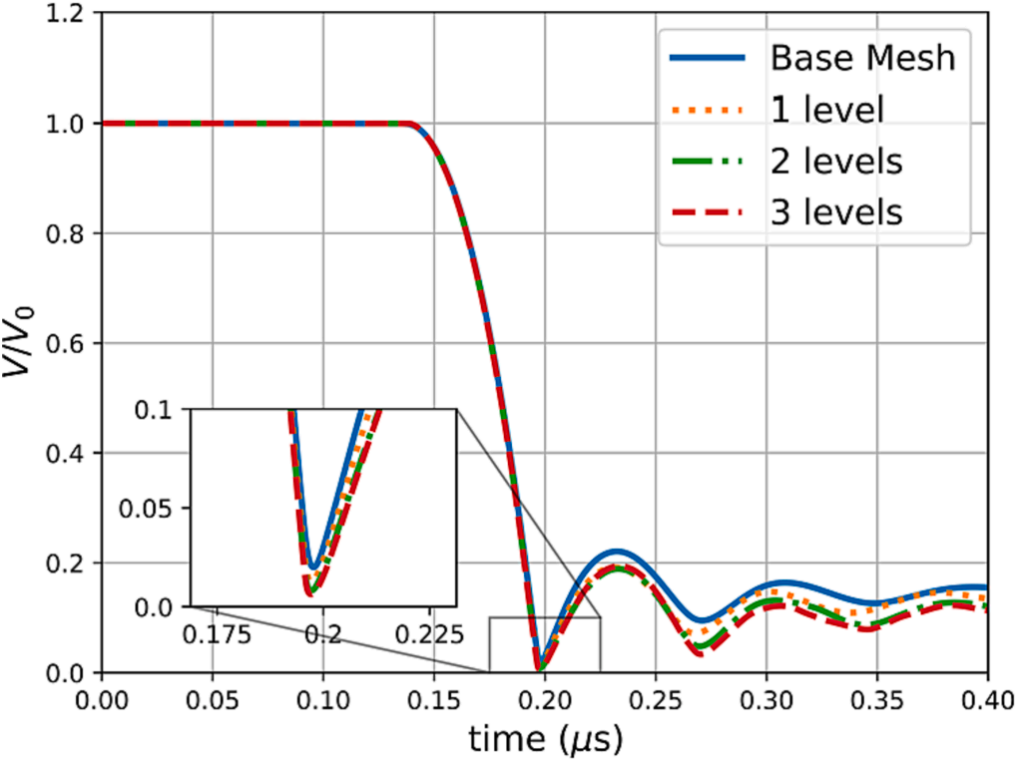
Mesh convergence study on the ultrasound-induced collapse of an air bubble of $R_{0}=10\mu m$ near bileduct. Temporal evolution of the normalized air volume for 4 different meshes.

## Appendix A Mesh convergence

A mesh convergence study on the ultrasound-induced collapse of an air bubble of $R_{0}=10\mu m$ near bileduct. The temporal evolution of the normalized air volume for 4 different meshes is shown in Fig. 16. The solution with two levels of adaptive mesh refinement provides satisfactory results. The mesh with up to three levels of adaptive mesh refinement was chosen for the added quality and sharpness of the contours.
